# Crash characteristics and patterns of injury among hospitalized motorised two-wheeled vehicle users in urban India

**DOI:** 10.1186/1471-2458-9-11

**Published:** 2009-01-12

**Authors:** Michael Fitzharris, Rakhi Dandona, G Anil Kumar, Lalit Dandona

**Affiliations:** 1 George Institute for International Health and School of Public Health, University of Sydney, Sydney, Australia; 2 Accident Research Centre, Monash University, Melbourne, Australia; 3 Accident Research Centre, Monash South Africa, Johanesburg, South Africa; 4 George Institute for International Health – India, Hyderabad, India; 5 Administrative Staff College of India, Hyderabad, India

## Abstract

**Background:**

Traffic crashes and consequent injuries represent a growing public health concern in India, particularly in light of increasing motorization. Motorised two-wheeled vehicles (MTV) constitute a large portion of the vehicle fleet in India. We report the crash characteristics and injury patterns among a cohort of MTV riders and pillions presenting to hospital post-crash.

**Methods:**

Consecutive MTV riders and pillions, whether alive or dead, injured in a road traffic crash presenting to the emergency departments of two government hospitals and three branches of a private hospital in urban Hyderabad, India, were recruited to this study.

**Results:**

378 MTV users were enrolled to the study of whom 333 (88.1%) were male, 252 (66.7%) were riders and median age was 31.3 years. A total of 223 (59%) MTV users were injured in multi-vehicle crashes while one-third had a frontal impact. The majority (77%) were assessed as having a Glasgow coma score (GCS) of 13–15, 12% a GCS of 9–12 and 11% a GCS of 3–8. No difference was seen in the severity distribution of injuries based on GCS among riders and pillions. Open wounds and superficial injuries to the head (69.3%) and upper extremity (27%) and lower extremity (24%) were the most common injuries. 43 (11%) sustained an intracranial injury, including 12 (28%) with associated fracture of the bones of the head. There were few differences in types of injuries sustained by riders and pillions though riders had a significantly lower risk of crush injuries of the lower extremity than pillions (relative risk, RR 0.25, 95% CI 0.08–0.81) and female pillions were at a significantly lower risk of sustaining fractures of the lower extremity than male pillions (RR 0.30, 95% CI 0.09 – 0.94). Overall, 42 (11%) MTV users died, of which 42.8% died before reaching the hospital. Only 74 (19.6%) MTV users had worn a helmet correctly and failure to wear a helmet was associated with a five times greater risk of intracranial injury (RR 4.99, 95% CI 1.23–20.1). Of the 19 pre-hospital deaths, 16 (84%) had not worn a helmet.

**Conclusion:**

Head injuries accounted for the major proportion of injuries sustained in MTV users. Non-helmet use was associated with increased risk of serious head injuries. The data presented on the nature and severity of injuries sustained by MTV users can assist with planning to deal with these consequences as well as prevention of these injuries given the high use of MTV in India.

## Background

India has experienced rapid growth in motorization in the last decade, with concomitant increases in road traffic injury (RTI) related mortality being observed.[1,2] Pedestrians, motorised two-wheeled vehicle (MTV) users, and cyclists are the most vulnerable road user groups in terms of injuries and fatalities resulting from road traffic crashes in India.[1,3-6] In actual numbers, over 105,700 people died and 452,900 were injured due to RTI in India in 2006 alone, with MTV users accounting for 17.8% of the fatalities.[7] Moreover, fatalities due to RTI in India are projected to increase by 150% by the year 2020,[5] with the majority of this increase being among users of MTV.[5,6] With MTV representing 70% of all vehicles registered in India in the year 2004 [8], the centrality of MTVs as a means of daily transport in India is clear.

The nature of injuries sustained in MTV crashes in high-income regions is well understood, [2] however, the characteristics of crashes and associated injuries sustained by MTV users in India are less well understood. In this paper, we report these data for MTV users from a prospective outcome study in an urban population from Hyderabad city in India, with specific emphasis on exploring differences, if any, between riders and pillions in injury outcomes. The annual incidence of non-fatal RTI as MTV user among 5–49 years age group is estimated at 6.3% in Hyderabad highlighting the enormous burden of RTI among MTV users.[9] MTV users accounted for one-third of the fatalities in road traffic crashes in Hyderabad in the year 2002.[4]

## Methods

The setting for the study was Hyderabad city in India. Hyderabad has a population of 3.8 million[10] and had 1.2 million registered motor-vehicles in 2001–2002, with the majority being MTV (77%).[8] This study was approved by the Ethics Committee of the Administrative Staff College of India, Hyderabad, India.

A total of 781 consecutive RTI cases reporting to two large public hospitals and three branches of a large private hospital in Hyderabad were recruited for this study from November 2005 to June 2006. People of all ages with RTI who reported to the emergency department or were brought in dead to these hospitals were included. RTI was defined as any injury resulting from road traffic crash irrespective of the severity and outcome. One person refused participation in the study.

### Interview process

Trained interviewers were posted round-the-clock in the emergency departments and mortuary to capture all consecutive RTI cases. They documented the contact address of all the RTI cases in detail to ensure that a follow-up interview was possible. Interviews were conducted using a questionnaire designed for this study after obtaining written informed consent from the injured person or the care-taker, or a responsible adult family member for those that had died. Data were collected from the injured person where possible, or the care-taker or adult family member where this was not possible.

Detailed data on the demographics of those injured, characteristics of the crash, helmet use for MTV users, driving under influence of alcohol, Glasgow coma score (GCS) [11] on arrival at hospital, details of injuries sustained, and final disposition were documented.

### Injury details

Details of injuries sustained were completed by the hospital physician posted in the emergency department who attended to the particular RTI case. For those who died in at the scene or en-route to the emergency department, a physician attached to the hospital mortuary documented the injuries sustained. The injuries were noted in detail and were later classified according to broad categories as per *International statistical classification of diseases and related health problems Version 10 *(ICD-10) [12] and the Abbreviated Injury Scale[13] by MF. The Injury Severity Score (ISS) score was derived for each case [14], and in addition, the highest AIS severity for each region (MAIS) was noted. The coding of injuries was not undertaken by the hospital physicians due to necessary training requirements. The medical notes were referred to when insufficient details of injuries were described to accurately ascribe an ICD or AIS code

### Data analysis

Data were entered into an MS Access database. The main outcomes reported are the crash characteristics, injury patterns and severity among the riders and pillion passengers of MTV. MTV types included mopeds (≤ 100 cc engine), scooterettes (60–105 cc engine), scooters (100–150 cc engine), and motor cycles (≥ 100 cc engine). Injuries were classified according to broad ICD-10 classifications. Superficial injuries of the head and neck, and thorax and abdomen were combined, with the same process for open wounds, fractures, and other unspecified injuries sustained for ease of analysis and presentation.

Patient and injury characteristics were examined for riders and pillion passengers, with comparisons made using chi-square tests, Fishers Exact Test, and relative risk (RR) ratios with 95% confidence intervals (CI) presented where appropriate.[15,16] Principal comparisons for the injury patterns were made between riders and pillions including helmet use and hospital mortality. The Mann-Whitney test, chi-square test and independent samples t-test was used to assess group differences where appropriate.[15,16] Logistic regression model was constructed to examine the predictors of mortality.[17] Analysis was conducted using STATA V.9.2 [18] and statistical significance was set at p ≤ 0.05.

## Results

Among the 781 RTI cases recruited to the study, 378 were MTV users (48.4%), 203 pedestrians (26%), 88 motorized three-wheeled vehicle occupants (11.3%), 36 occupants of cars and jeeps (4.6%), 41 occupants of buses and trucks (5.2%), and 35 cyclists (4.5%).

### Rider and pillion characteristics

In the study recruitment period, there were 378 MTV users recruited to the study with 252 (66.7%) being riders. Motorcycles were the most common type of MTV (56.1%) followed by scooters (38.6%), and mopeds (5.3%) with no difference in vehicle type between riders and pillions, (χ^2^(1) = 0.9, p ≥ 0.05) (Table 1). There was, however, a significant sex difference between riders and pillions, with 245 (97.2%) of riders being male as compared to 88 (69.8%) of pillions being male (χ^2^(1) = 60.0, p ≤ 0.05). The median age for riders was significantly older than that of the pillions (z = 3.9, p ≤ 0.05). There were significantly fewer riders < 20 years of age (11.1%) than pillions (26.2%) and more riders (29%) > 40 years of age than pillions (18%) (χ^2^(2) = 15.7, p ≤ 0.05). There were few children aged 1–9 years (3, 0.8%) and 10–14 years of age (9, 2.4%), with all but one child aged 10 – 14 years being pillions. There was no significant difference in the distribution of marital status or educational attainment between riders and pillions.

**Table 1:** Demographic characteristics of injured riders and pillions

**Characteristics**	**Rider** **(n = 252, 66.7%)**	**Pillion** **(n = 126, 33.3%)**	**Total** **(n = 378, 100%)**
Road-user type (%)			

Moped	14 (5.6%)	6 (4.8%)	20 (5.3%)

Scooter	101 (40.1%)	45 (35.7%)	146 (38.6%)

Motorcycle	137 (50.4%)	75 (59.5%)	212 (56.1%)

Male (%)	245 (97.2%)	88 (69.8%)	333 (88.1%)

Age (years)			

Mean (Standard Deviation)	32.8 (11.5)	28.3 (12.7)	31.3 (12.1)

Median, Range	30, 14–68	25, 4–70	28 (4–70)

### Crash characteristics

Of the 378 MTV riders and pillions, 155 (41%) were injured as a result of a single vehicle crash (SVC) while 223 (59%) were injured as a result of a multi-vehicle crash (MVC) (Table 2). There was no difference in the proportion of SVC and MVC between riders and pillions (χ^2^(1) = 1.07, p ≥ 0.05). There were few differences in collision partners for multiple vehicle crashes. One-third of riders and pillions (36%) had a frontal impact, with an additional 8% impacting the rear of an opposing vehicle, 25% were struck from behind, and 27.6% were struck from the side.

**Table 2:** Crash characteristics of injured riders and pillions

**Characteristics**	**Rider** **(n = 252, 66.7%)**	**Pillion** **(n = 126, 33.3%)**	**Total** **(n = 378, 100%)**
Type of crash and collision partner			

*Single vehicle*	*108 (42.9%)*	*47 (37.3%)*	*155 (41%)*

Vehicle skidded	57 (52.8%)	13 (27.7%)	70 (45.2%)

Hit a non-moving object	44 (40.7%)	21 (44.7%)	65 (41.9%)

Fall from vehicle	3 (2.8%)	12 (25.5%)	15 (9.7%)

Collision with animal/others	4 (3.7%)	1 (2.1%)	5 (2.6%)

*Multiple vehicle crash*	*144 (57.1%)*	*79 (62.7%)*	*223 (59%)*

Bus/heavy vehicle	52 (36.1%)	28 (35.4%)	80 (35.9%)

Car/Jeep	35 (24.3%)	26 (32.9%)	61 (27.4%)

Motorised two-wheeled vehicle	32 (22.2%)	14 (17.7%)	46 (20.6%)

Motorised three-wheeled vehicle	15 (10.4%)	10 (12.7%)	25 (11.2%)

Pedestrian/Cyclist/Other	10 (6.9%)	1 (1.3%)	11 (4.9%)

Time of crash			

00:00 – 05:59	53 (21%)	26 (20.6%)	79 (20.9%)

06:00 – 11:59	24 (9.5%)	15 (11.9%)	39 (10.3%)

12:00 – 17:59	66 (26.2%)	42 (33.3%)	108 (28.6%)

18:00 – 24:00	109 (43.3%)	43 (34.1%)	152 (40.2%)

Alcohol use*			

Yes, confirmed in ED	35 (13.9%)	13 (10.3%)	48 (12.7%)

Suspected in ED	5 (2.0%)	1 (0.8%)	6 (1.6%)

No, confirmed in ED	23 (9.1%)	11 (8.7%)	34 (9.0%)

Not checked in ED	189 (75%)	101 (80.2%)	290 (76.7%)

*ED: emergency department

Alcohol content was not assessed for 76.7% of riders and pillions, however where assessed in the Emergency Department, there was no difference in the proportion of riders (55.6%) and pillions (52%) with the presence of alcohol confirmed (χ^2^(1) = 0.7, p ≥ 0.05); the blood alcohol content level was not specified.

### Injury severity and hospitalization details

Of the 378 MTV riders and pillions, 6 (1.6%) died at the scene, 12 (3.2%) died en-route to hospital, and 360 (95.2%) arrived alive at hospital, of whom 24 (6.3%) later died in hospital (18 riders, 6 pillions). In total, 42 (11.1%) died as a consequence of injuries sustained in the crash, including 29 riders (11.5%) and 13 pillions (10.3%), and hence the proportion of survivors was 89%. The in-hospital mortality rate was 6.6% (24 of 360). There was no difference between riders and pillions with respect to fatality-survival outcome (χ^2^(1) = 0.1, p ≥ 0.05).

Table 3 presents a range of parameters for those presenting alive to hospital. No difference was seen in the severity distribution of injuries based on the mean and median ISS between riders and pillions with 8.6% sustaining an ISS > 15 (p ≥ 0.05). There was a significant difference (p ≤ 0.05) in the mean and median ISS for those who died (mean 20.7, SD 27.1; median 10; range 1 – 75) and those who survived (mean 4.5, SD 4.9; median 2; range 1 – 25). Approximately 10% of the cases had an ISS > 15, thereby being classified as major trauma. Seven cases had ISS score of 75, and in each case sustained an AIS 6 (maximum) injury to the head. There was no significant difference in GCS between riders and pillions (p ≥ 0.05).

**Table 3:** Severity parameters and length of stay for riders and pillions presenting alive to hospital

	**Rider** **(n = 241, 67%)**	**Pillion** **(n = 119, 33%)**	**Total** **(n = 360, 100%)**
Patient discharge status			

Alive	223 (92.5%)	113 (95%)	336 (93.3%)

Died	18 (7.5%)	6 (5%)	24 (6.7%)

Injury Severity Score (ISS)			

Mean (SD)	5.1 (7.2)	5.4 (8.0)	5.2 (7.5)

Median	2	4	2

Range	1 – 75	1 – 75	1 – 75

MAIS*			

Minor (1)	122 (50.6%)	55 (46.2%)	177 (49.2%)

Moderate (2)	57 (23.7%)	28 (23.5%)	85 (23.6%)

Serious (3)	32 (13.3%)	24 (20.2%)	56 (15.6%)

Severe (4)	19 (7.9%)	6 (5.0%)	25 (6.9%)

Critical (5)	3 (1.2%)	0 (0%)	3 (0.8%)

Maximum (6)	1 (0.4%)	1 (0.8%)	2 (0.6%)

Unknown	7 (2.9)	5 (4.2%)	12 (3.3%)

Glasgow Coma Score (GCS) on arrival at hospital†			

13–15	185 (76.8%)	91 (76.5%)	276 (76.7%)

9–12	29 (12.0%)	14 (11.8%)	43 (11.9%)

3–8	27 (11.2%)	14 (11.8%)	41 (11.4%)

*Highest AIS severity for each region

†GCS 13 – 15 indicates mild or no traumatic brain injury, GCS 9 – 12 moderate injury and GCS 3 – 8 severe injury

Among the 372 post-crash survivors (excluding deaths at scene), 176 (47.3%) reported a loss of consciousness (LOC), though data on the severity and duration of LOC were not collected; there was, however, no difference in the proportion of riders and pillions with a reported LOC (χ^2^(3) = 0.03, p = 0.8). Notably though, of the riders (8) and pillions (4) who had died en-route to hospital, all were reported to have sustained a LOC indicating the presence of severe head injuries. Overall, the mean length of stay in the hospital (6.3 days, SD: 8.5 days; median: 3 days) was similar for riders and pillions (t(194.3) = 1.05, ≥ 0.05) as was the overall distribution (χ^2^(4) = 7.2, ≥ 0.05), with approximately 23% of riders and 29% of pillions being admitted for 8 days or longer.

### Injuries sustained

The most common injuries sustained were open wounds to the head-neck such as lacerations (35%), followed by superficial wounds of the head-neck (34%), the upper extremity (26.7%), and the lower extremity (24%) (Table 4). Approximately 12% of the riders and pillions sustained open wounds of the lower extremity, and 6.6% sustained superficial wounds of the thorax and abdomen regions, and 6% sustained open wounds of the upper extremity. It is evident that superficial and open wounds are by and large the most common type of injuries sustained by these riders and pillions. Fractures of the lower extremity were sustained by one-fifth (72, 19%) of riders and pillions. The most common factures of the lower extremity were of the lower leg including ankle (54, 14%), the femur (21, 5.6%), and the bones of the foot (1, 0.3%).

**Table 4:** Injury patterns and relative injury risk for riders and pillions

ICD-10 body region*	**Rider** **(n = 252)**	**Pillion** **(n = 126)**	**Relative risk (RR) of injury (referent: pillions)**	
	
	**N (%) injured**	**N (%) injured**	**RR**	**95% CI**^†^
Head and Neck				

Superficial	93 (36.9%)	35 (27.8%)	1.33	0.96–1.84

Open wound	95 (37.7%)	39 (31.0%)	1.22	0.89–1.65

Fracture	26 (10.3%)	13 (10.3%)	1.0	0.53–1.87

Intracranial	29 (11.5%)	14 (11.1%)	1.03	0.57–1.89

Crush	5 (2.0%)	2 (1.6%)	1.25	0.24–6.35

Traumatic amputation	1 (0.4%)	1 (0.8%)	0.50	0.03–7.92

Other unspecified	23 (9.1%)	13 (10.3%)	0.88	0.46–1.68

Neck, injury nerves	1 (0.4%)	0 (0.0%)	-	-

Thorax and Abdomen				

Superficial	19 (7.5%)	6 (4.8%)	1.58	0.65–3.86

Open wound	3 (1.2%)	2 (1.6%)	0.75	0.13–4.43

Fracture	13 (5.2%)	3 (2.4%)	2.17	0.63–7.46

Intra-thoracic organ, unspecified	1 (0.4%)	0 (0.0%)	-	-

Intra-abdominal organs, injury	2 (0.8%)	2 (1.6%)	0.50	0.07–3.50

Lumbar spine/pelvis: dislocation, sprain	1 (0.4%)	0 (0.0%)	-	-

Other unspecified	11 (4.4%)	6 (4.8%)	0.92	0.35–2.42

Upper Extremity				

Superficial	72 (28.6%)	29 (23.0%)	1.24	0.85–1.80

Open wound	18 (7.1%)	4 (3.2%)	2.25	0.78–6.51

Fracture	25 (9.9%)	12 (9.5%)	1.04	0.54–2.00

Dislocation, sprain, joints & ligaments	1 (0.4%)	0 (0.0%)	-	-

Crush	0 (0.0%)	2 (1.6%)	-	-

Other unspecified	13 (5.2%)	4 (3.2%)	1.65	0.54–4.88

Lower Extremity				

Superficial	56 (22.2%)	34 (27.0%)	0.82	0.57–1.19

Open wound	24 (9.5%)	20 (15.9%)	0.60	0.35–1.04

Fracture	46 (18.3%)	26 (20.6%)	0.88	0.57–1.36

Dislocation, sprain, joints & ligaments	1 (0.4%)	0 (0.0%)	-	-

Crush	4 (1.6%)	8 (6.3%)	0.25	0.08–0.81

Other unspecified	5 (2.0%)	7 (5.6%)	0.35	0.11–1.10

*ICD: International statistical classification of diseases and related health problems Version 10

†CI: Confidence interval

Of note was that 43 (11%) had sustained an intracranial injury with 12 (28%) also sustaining an associated fracture(s) of the bones of the head. Intracranial injuries included for example, concussion, subdural haemorrhage, and subarachnoid haemorrhage. In addition to the 14 riders and pillions who had sustained both an intracranial injury and a fracture of the bones of the head, a further 25 riders and pillions sustained a fracture to this region but without intracranial injuries. The risk of injury among riders and pillions differed only for one injury subtype, with the risk of crush injuries of the lower extremity being lower for riders (1.6%) than for pillions (6.3%) (RR 0.25, 95% CI 0.08–0.81).

We also explored potential differences in injury risk for male pillions (n = 88) and female pillions (n = 38) given the propensity for female pillions to sit sideways across the seat rather than facing forward. Interestingly, female pillions (7.9%) were at a significantly lower risk of sustaining fractures of the lower extremity than male pillions (26%) (RR 0.30, 95% CI 0.09 – 0.94). Female pillions (18%) were, however, somewhat more likely than male pillions (6.8%) to sustain fractures of the head and neck region (RR 2.7, 95% CI 0.97 – 7.49), and 8% of female pillions sustained open wounds of the upper extremity compared to only 1% of male pillions (Fishers Exact p = 0.09).

### Helmet use and head injury severity

Among the 378 MTV riders and pillions, only 74 (19.6%) wore a helmet correctly (20% open faced; 80% closed faced) (Table 5). Riders (29%) were significantly more likely than pillions (n = 1, 0.8%) to wear a helmet (RR 36.5, 95% CI 5.1 – 259.5). On examination of non-wearers, a small proportion (9, 2.4%) were found to have worn the helmet but had failed to fasten the strap, while another 27 (7%) had carried a helmet at the time of crash. Excluding the 6 riders and pillions who died at the scene, 149 (49.8%) of the 299 non-helmet MTV users sustained an LOC while only 27 (37%) of the 73 wearing a helmet sustained an LOC (RR 1.34, 95% CI 0.98 – 1.85, p = 0.05). Considering only those 165 cases where the GCS was known, 95% (all but 1 patient) in each of the GCS 3–8 and GCS 9–12 categories were not wearing a helmet in contrast to 71% in the GCS 13–15 category. The risk of sustaining a moderate-severe head injury not wearing a helmet was 5 times higher than had a helmet been worn (27% vs 5.2%, RR 5.26, 95% CI 1.32 – 20.9).

**Table 5:** Helmet use among riders and pillions

**Helmet use**	**Rider (n = 252)**	**Pillion (n = 126)**	**All (n = 378)**
	**N (%)**	**N (%)**	**N (%)**

Used, Properly secured	73 (29.0%)	1 (0.8%)	74 (19.6%)

Not used			

Not properly secured	8 (3.2%)	1 (0.8%)	9 (2.4%)

Available, not used (carrying)	24 (9.5%)	3 (2.4%)	27 (7.1%)

Not available	147 (58.3%)	121 (96.0%)	268 (70.9%)

*Sub-total not wearing a helmet*	*179 (71%)*	*125 (99.2%)*	*304 (80.4%)*

Total	100%	100%	100%

In all head injury sub-types, the proportion of sustaining that injury was greater among non-helmet users than for those using helmets; this effect is most prominent for intracranial injuries and open wounds of the head (Table 6). More specifically, helmet non-use was seen to be associated with a 1.9 times higher risk of sustaining an open wound (RR 1.91, 95% CI 1.19–3.07, p = 0.002) and a 5 times higher risk of intracranial injury (RR 4.99, 95% CI 1.23–20.1, p = 0.004, Fishers Exact), with only two helmeted participants (both riders) sustaining an intracranial injury in contrast to 41 non-helmeted participants. Intracranial injuries were examined more closely. Focal brain injury (S06.3; n = 13, 30%) was the most common, followed by sub-dural hematoma (SDH, S06.5; n = 10, 23%), sub-arachnoids haemorrhage (SAH; S06.6; n = 9, 21%), concussion (S06.0; n = 3, 7%), traumatic cerebral odema (S06.1; n = 2, 5%), extra-dural hematoma (S06.4; n = 2, 5%), while four sustained injuries of such severity that they were classified as intracranial injury – other (S06.9); all four died. Of the 10 participants sustaining SDH, two were helmeted riders in contrast to eight (5 riders, 3 pillions) non-helmeted participants.

**Table 6:** ICD-10 coded head injuries and helmet use and relative risk of injury

ICD-10 Head Injury*	**Helmet worn (n = 74)**	**Helmet not worn (n = 304)**	**Not worn relative risk (RR) of injury to worn**	
	
	N (%)	N (%)	RR	95% confidence interval
Superficial	22 (29.7%)	106 (34.9%)	1.17	0.80–1.72

Open wound	16 (20.3%)	118 (38.8%)	1.91	1.19–3.07

Fracture	6 (6.8%)	33 (10.5%)	1.56	0.63–3.86

Intracranial	2 (2.7%)	41 (13.5%)	4.99	1.23–20.1

Crush	1 (1.4%)	6 (2.0%)	1.46	0.17–11.9

Traumatic amputation	0 (0%)	2 (0.7%)	-	-

Other unspecified	5 (6.8%)	31 (10.2%)	1.51	0.61–3.74

*ICD: International statistical classification of diseases and related health problems Version 10

In total, 36 (11.8%) of 304 non-helmet wearers died compared to 6 (8.1%) of 74 helmet wearers, indicating a higher mortality risk, however, this was not statistically significant (RR 1.46, 95% CI 0.46 – 3.33, p ≥ 0.05). Notably of the 19 pre-hospital deaths, 16 (84%) were not wearing a helmet.

### Association between injuries and mortality

As noted, 42 MTV users in the study died. The most notable injury types among the MTV riders (29, 69%) and pillions (13, 31%) who had died were open wounds of the head-neck (40%), intracranial injuries (31%) followed by crush injuries of the head (16.7%), and fractures of the head-neck (14.3%). Other notable injuries sustained among those who died were thoracic fractures (9.5%) and intra-abdominal organ injuries (9.5%), while the incidence of fractures of the lower extremity among those who died was low (2.4%, n = 1).

Given the association between helmet use and intracranial injuries noted above, we examined the relationship between intracranial injuries and mortality. Overall, 11% of riders and pillions sustained an intracranial injury, and of these 30% died; the mortality rate for those without an intracranial injury was 8.7%. Consequently, a univariate analysis of the relationship between intracranial injury and mortality indicated a mortality risk 3.5 times higher among those with an intracranial injury than those without s (RR 3.46, 95% CI 1.96 – 6.10). This relationship remained evident even with age, sex, and collision partner are entered into a multivariate logistic regression model (OR 5.97, 95% CI 1.4 – 25.1), and these additional parameters not being statistically significant predictors of intracranial injuries.

To further examine the association between injury type and mortality, a logistic regression model was developed with helmet use, age, sex, MAIS scores for the head, chest and abdomen, position (rider vs. pillion) and striking object 'forced' into the model given the focus of this paper (Table 7). Sixteen cases were excluded from the model due to unknown AIS/ISS scores (4%), resulting in a loss of 7 of the 42 deceased cases and 9 of the 336 cases discharged alive. Head injury MAIS, chest MAIS, abdomen-pelvis MAIS and being struck by a vehicle in the bus, truck, van category (relative to a single vehicle crash) were all significant predictors of mortality, while helmet use, position, and a number of striking object parameters were not statistically significant (Table 7). There was no interaction between head MAIS and helmet use. The model correctly classified 93% of cases, albeit with low sensitivity (31%) but extremely high specificity (98.5%), with an Area under the Curve of 0.85, demonstrating excellent discrimination.[17]

**Table 7:** Association of mortality with select variables using multiple logistic regression

**Parameter**	**Outcome**	**Referent**	**Odds ratio**	**95% confidence interval**		**P**
					
				**Upper**	**Lower**	
Sex	Female	Male	1.50	0.35	6.55	0.6

Helmet use	Not Worn	Worn	0.60	0.19	1.92	0.4

Position	Pillion	Rider	0.86	0.28	2.67	0.8
	
Collision type	Others including pedestrians	Single vehicle	2.07	0.65	6.60	0.2
	
	Car	Single vehicle	0.75	0.17	3.22	0.7

	Bus, truck, van	Single vehicle	3.43	1.25	9.45	0.02

MAIS – Head*	*Continuous, 0 – 6*		2.03	1.58	2.61	< 0.01

MAIS – Chest*	*Continuous, 0 – 6*		2.03	1.05	3.93	0.04

MAIS – Abdomen*	*Continuous, 0 – 6*		3.72	1.80	7.86	< 0.01

Age	*Continuous*		1.02	0.98	1.05	0.3

*MAIS is the highest AIS severity by body region

## Discussion

This study reports on the nature of crashes and patterns of injuries sustained among a cohort of consecutive riders and pillions of MTV presenting to the emergency departments post-crash in urban India. Overall, 59% of MTV riders and pillions were injured in multiple vehicle crashes, with 40% occurring in the evening and a further 21% between midnight and 6 am, a pattern typical of MTV crashes.[19-22] Collision partners for those involved in multiple vehicle crashes tended to be large vehicles such as buses and trucks. Single vehicle crashes were dominated by vehicles skidding, presumably following avoidance maneuvers and loss of control, and striking fixed roadside objects. The demographic and crash profile of riders and pillions reported here are similar to those reported from India previously and from elsewhere.[1-4,19,20,23]

The findings indicate few differences between MTV riders and pillions in the type of injuries sustained. These differences were limited to pillions sustaining a higher proportion of crush injuries, however, the actual numbers of riders and pillions with crush injuries was low. While there were few notable differences in injury risk between riders and pillions, we report that female pillions are at a somewhat greater risk of fractures of the head and neck than their male counterparts, and at a similarly higher risk of open wound upper extremity injuries. As a group, female pillions sustained fewer lower extremity injuries than male pillions. This result may be associated with the increased propensity for female pillions to sit sideways on cycle and MTV for reasons of comfort mainly due to the Indian way of dressing.[3] From a biomechanics point of view, this may be associated with an increased risk of being 'thrown off' or falling from the vehicle, with the consequence of the outstretched arm and head-neck complex bearing much of the force. It is notable here that females represented 30% of pillions in contrast to 2.8% of riders, and only one pillion was wearing a helmet. This finding is clearly of concern, and highlights the need for appropriate seating and increased helmet use among female pillions.

The most commonly sustained injuries were to the head followed by injuries of the lower and upper extremity as reported previously from other populations.[1,2,24-28] The mortality rate was 11%, a figure comparable with that reported elsewhere for motorcyclists.[19] While helmet use was not directly associated with mortality in this sample, there was a strong association between helmet use and intracranial injuries, and in addition a strong association between intracranial injuries and head injury severity and mortality. These latter two findings, in particular, are concerning given that the overall helmet wearing rate among MTV riders and pillions was low (19.6%) in this study population. This low rate of helmet use among MTV users in India has been documented previously.[1,3,29] The increased risk of serious head injury in the absence of helmet wearing as has been documented previously and our study reinforces these findings in a developing world context.[30-34] The combined findings of a significantly higher risk of intracranial injuries for non-helmet users and the relationship between head injury severity and mortality demonstrates an urgent need for increasing helmet use in India. While this study provides evidence of the benefits of helmet use in mitigating serious intracranial injuries, earlier studies have reported reductions in head-injury associated mortality through the use of helmets.[2,3,24,35-39] It remains important that helmets used are of standard quality with respect to protection, as inferior, non-standard helmets have been shown to offer little useful protection in the event of a crash.[30]

Injuries to thorax and abdominal regions were also found to be associated with a higher risk of mortality in our study. Such association has also been previously reported from a large sample of motorcycle crashes in USA.[40] These findings suggest the importance of protecting these vital organs in addition to the head in motorcycle crashes and therefore the need for protective clothing in addition to helmets to reduce the mortality burden in this population.[19,20,41] Information on protective clothing was not collected in our study as the concept of such clothing does not exist in our population.

Importantly, most MTV riders and pillions (75%) were not tested for alcohol use, and when tested around 55% tested positive to alcohol, however the blood alcohol content was not recorded. These figures pertaining to alcohol use are difficult to interpret as bias in testing may be a factor in the high proportion of confirmed cases. In a study of traumatic brain injuries from south India which examined the role of alcohol, RTI incidence was found to be similar among alcohol users and non-users, however, MTV users and pedestrians were involved in crashes to a greater extent among the alcohol users and the severity of brain injuries, duration of hospital stay, death and post-traumatic disabilities were significantly higher among alcohol users compared with non-users.[42] One of the critical steps to reduce burden of RTI and the resulting mortality is to have reasonable evidence on the risk factors. The role of alcohol in RTI is well-documented in the developed countries and relevant road safety intervention programmes are in place.[2] Our data highlight the urgent need for proper documentation of alcohol use in RTI in India so that strategies and mechanisms to reduce the related burden can be addressed.[1,2,43]

This study has a few limitations. Injuries were coded using the information entered into the study database text fields from the study questionnaire rather than directly from the medical record, though the medical record and autopsy record formed the basis of the information documented in the study questionnaire. Where there was any doubt as to the nature of the injury these were queried and clarified by consultation with the medical record; this occurred in all 13 cases where the description of the injury was recorded as 'bleeding from the ears', given the possible differences in injury descriptors. While every effort was made to code the injuries accurately, it is possible for some bias to exist as the ICD and AIS/ISS coding. This is particularly true for fatalities pre-hospital, as 7 cases had an ISS < 9, 2 between 10 and 17, 5 with an ISS of 75, and in 4 cases there was insufficient, detailed and precise information to calculate an ISS score. However, this affected only a relatively small number of cases. In addition, where AIS codes were allocated, there was considerable use of 'Injury Not Further Specified' (NFS) codes, hence it is likely the ISS scores for the cases are conservative.

A further limitation is that we did not collect information from the first hospital the patient attended, and it is possible that this would bias the interpretation of injuries sustained, particularly the objective assessment of head injury severity immediately post-crash, hence we chose only to report the GCS for those attending the hospital directly from the scene.

Despite these limitations, the data presented in this paper provide important information on the nature and severity of injuries sustained by MTV users in India that can assist with planning to deal with these consequences as well as prevention of these injuries.

## Conclusion

This study reports on the nature of injuries sustained and associated helmet non-use among MTV riders and pillions presenting to emergency department post-crash in urban India. There were few but notable differences in the overall injury pattern between MTV riders and pillions, and between male and female pillions. Head injuries were the most common type of injury sustained and helmet use was low, highlighting the need for increasing helmet use given the high use of MTV in India and its projected increase.

## Abbreviations

CI: Confidence interval; ED: emergency department; GCS: glasgow coma scale; LOC: loss of consciousness; ISS: injury severity score; MAIS: highest AIS severity for each region; MTV: motorised two-wheeled vehicles; MVC: multiple vehicle crash; RR: relative risk; RTI: road traffic injury; SVC: single vehicle crash; ICD-10: The International Statistical Classification of Diseases and Related Health Problems 10th Revision.

## Competing interests

The authors declare that they have no competing interests.

## Authors' contributions

MF planned and led the drafting of the manuscript in conjunction with RD, coded the injuries with the assistance of GAK, and undertook the analysis. RD conceptualized and designed the study, GAK managed the data, LD contributed to the concept and design of the study. All authors were involved in the interpretations of the findings and drafting of the manuscript. RD is the guarantor for the study.

## Pre-publication history

The pre-publication history for this paper can be accessed here:


